# Gastrointestinal parasitism of goats in hilly region of Meghalaya, India

**DOI:** 10.14202/vetworld.2017.81-85

**Published:** 2017-01-19

**Authors:** Meena Das, R. Laha, A. Goswami, A. Goswami

**Affiliations:** Division of Animal Health, ICAR Research Complex for NEH Region, Umiam - 793 103, Meghalaya, India

**Keywords:** gastrointestinal parasites, goat, helminths, Meghalaya, protozoa

## Abstract

**Aim:**

The aim of this study was to determine the prevalence of gastrointestinal (GI) parasitic infections in goats of hilly region of Meghalaya.

**Materials and Methods:**

A total of 834 fecal samples of goats were screened for 1 year (2014-2015) using flotation techniques.

**Results:**

The overall prevalence of GI parasitic infections in goats was 28.65%. Season-wise highest infections were recorded during rainy season (34.92%) followed by cool (26.87%), hot (26.62%), and cold (20.39%) seasons. Helminths and protozoa infections were recorded in 63.60% and 23.02% animals, respectively. Among the helminths, *Strongyle* spp. (32.63%) was recorded highest followed by *Trichuris* spp. (12.55%), *Moniezia* spp. (10.04%), and *Trichuris* spp. (8.36%). Among protozoa, only *Eimeria* spp. was detected. Seven different species of *Eimeria* spp. were identified, viz., *Eimeria christenseni*, *Eimeria hirci*, *Eimeria caprina*, *Eimeria jolchijevi*, *Eimeria ninakohlyakimovae*, *Eimeria arloingi*, and *Eimeria kocharii* for the first time from Meghalaya. Maximum egg per gram and oocyst per gram of feces were recorded in the month of August (932.4) and September (674.05), respectively. Mixed infections were recorded in 13.38% samples. Coproculture of goat fecal samples revealed the presence of *Haemonchus contortus* (72.16%), *Oesophagostomum* spp. (14.41%), *Strongyloides* spp. (8.91%), and *Trichostrongylus* spp. (4.50%) larvae.

**Conclusion:**

This study indicates that GI helminths and protozoa infections are prevalent in goats of this hilly region of Meghalaya, throughout the year and highly prevalent during rainy season.

## Introduction

Domestic goat is among the earliest animals domesticated by man and is distributed worldwide with higher concentrations in tropical areas and in dry zones [1]. Goats are excellent meat producers for human consumption in view of its short generation intervals and the absence of religious taboos associated with their meat as they are rich sources of protein and can help bridge the gap of protein malnutrition among consumers [2]. Gastrointestinal (GI) parasitic infections are common in goats causing considerable economic losses as a consequence of mortality in infected animals and reduced weight gain. Most common GI parasites in goats are helminths and coccidia [3,4]. Coccidiosis in small ruminants is caused by a protozoan parasite which belongs to genus *Eimeria* and often can be seen in small as well as in large intestine [5].

It leads to poor growth rate, diarrhea, dysentery, and anemia and supposed to be one of the economically most important diseases of small ruminants (sheep and goats) as far as intensive farming is concerned [6]. It is mainly suspected when animals are kept under poor hygienic conditions and the mortality is mainly evident during weaning period [5]. These parasites often lead to destruction of epithelial cells of intestine and interfere with intestinal microflora [7]. 17 *Eimeria* species have been described in goats [8]. However, only nine *Eimeria* species could create an infection and the most pathogenic species in goat is *E. arloingi* [9]. According to Dik [10] and Nourani *et al*. [11], *Eimeria* species from goat are also localized in liver, gallbladder, bile ducts, hepatic and mesenteric lymph nodes as well as small and large intestine. *E. arloingi* causes polyp and nodular hyperplasia in intestinal mucosa [9] and could result in fatal coccidiosis. In goats, the most important species which leads to clinical signs includes *E. ninakohlyakimovae* and *E. arloingi* [12,13]. Environmental factors also play a vital role in the parasitic infections and the prevalence rate of infections may vary [14]. The disease is mostly transmitted by ingestion of contaminated feed, water, pastures with parasitic eggs, ova, cysts, etc.

Therefore, taking into account the significance of the GI parasites as one of the most important causes of economic losses, the present study was designed to determine the prevalence of GI parasites in goats of hilly region of Meghalaya.

## Materials and Methods

### Ethical approval

The experiments comply with the guidelines laid down by the Institutional Ethical Committee and in accordance with the country law.

### Study area

The present study was conducted in two districts of Meghalaya, *viz*., Ri Bhoi and East Khasi Hills. Ri Bhoi district occupies an area of 2378 km^2^ and lies between 25°15’ and 26°15’ North latitudes and 91°45’ and 92°15’ East longitudes. This district is characterized by rugged and irregular land surface. It includes a series of hill ranges which gradually sloped toward the north and finally joins the Brahmaputra Valley (https://en.wikipedia.org/wiki/Ri-Bhoi_district). East Khasi Hills district forms a central part of Meghalaya and covers a total geographical area of 2748 km^2^. It lies between 25°07’ and 25°41’ North latitudes and 91°21’ and 92°09’ East longitudes. This district is mostly hilly with deep gorges and ravines on the southern portion (https://en.wikipedia.org/wiki/East_Khasi_Hills_district). The study area was situated at an altitude of about 600-1800 m above mean sea level where average monthly minimum and maximum temperature were 6.5°C and 30.8°C, respectively. The average monthly relative humidity prevailed during the study period was 61.6% (minimum) to 88.9% (maximum) with average annual total rainfall 2877 mm.

### Study period

The study was conducted for one calendar year from April 2014 to March 2015 and divided into four seasons, *viz*., hot (March, April), rainy (May, June, July, August, September), cool (October, November), and cold (December, January, February).

### Study method

A total of 834 fecal samples of goats were collected from different places of Ri Bhoi and East Khasi Hills districts of Meghalaya. Fecal samples were collected directly from the rectum of the different animals at monthly intervals. The age of the animals ranged from 2 months to 1 year. All samples were kept in marked plastic pouch/vials. To find out the eggs/ova/cyst of helminths and protozoa, samples were examined by flotation and sedimentation techniques [15]. Samples not being examined on the same day were preserved and stored at refrigerated temperature (4°C) for next day examination. The egg per gram (EPG) and oocyst per gram (OPG) of feces were estimated by modified McMaster technique. Fecal samples found positive for *Strongyle* group of parasites were subjected to coproculture for obtaining third stage infective larvae (L_3_). The pooled fecal samples were finely broken and mixed with sufficient quantity of activated charcoal. The mixture was then packed loosely in glass culture dishes and incubated at 27°C for 7 days as per the described procedure in MAFF [15]. The L_3_ was subsequently harvested and identified according to Borgsteede and Hendriks [16] and Soulsby [17]. Sporulated oocysts of *Eimeria* spp. were obtained by mixing feces containing oocyst of *Eimeria* spp. with 2.5% potassium dichromate solution as per the procedure described by Bhatia [18].

## Results and Discussion

**Figure-1:**
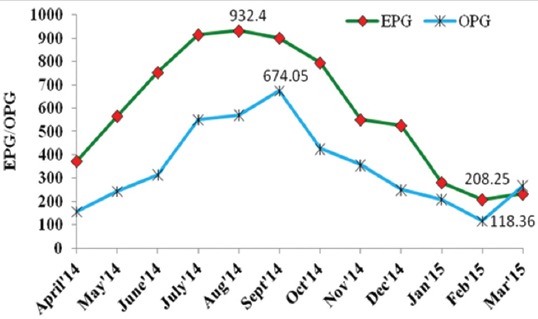
Month-wise intensity of egg per gram and oocyst per gram in goats of Meghalaya.

**Figure-2:**
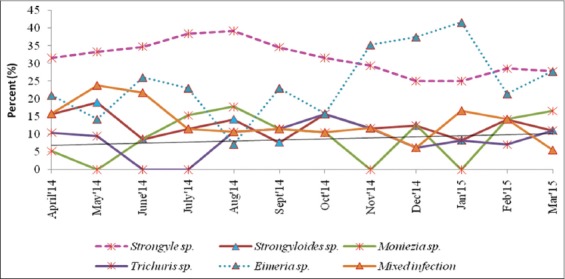
Percent prevalence of different species of gastrointestinal parasites in goats of Meghalaya.

**Figure-3:**
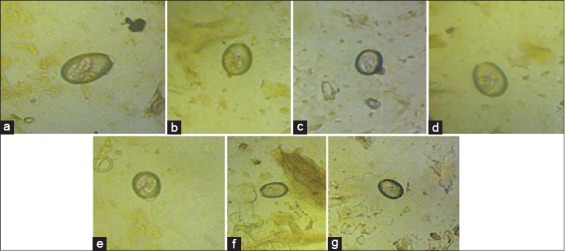
Different species of *Eimeria* spp. in goats of Meghalaya. (a) *Eimeria christenseni*, (b) *Eimeria jolchijevi*, (c) *Eimeria ninakohlyakimovae*, (d) *Eimeria caprina*, (e) *Eimeria hirci*, (f) *Eimeria kocharii*, (g) *Eimeria arloingi*.

Examinations of fecal samples revealed the prevalence of GI parasitic infections in goats of hilly region of Meghalaya, throughout the year. The overall prevalence of GI parasitic infections in goats was 28.65%. Season-wise highest infections were recorded during rainy season (34.92%) followed by cool (26.86%), hot (26.62%), and cold (20.39%) seasons (Table-1). Helminths and protozoa infections were recorded in 63.60% and 23.02% animals, respectively. Among the helminths, *Strongyle* spp. (32.63%) was recorded highest followed by *Strongyloides* spp. (12.55%), *Moniezia* spp. (10.04%), and *Trichuris* spp. (8.36%). Among protozoa, only *Eimeria* spp. (23.02%) was detected. Month-wise intensity of *Strongyle* spp. EPG and *Eimeria* spp. OPG of feces are shown in Figure-1. Maximum and minimum EPG was recorded in the month of August (932.4) and February (208.25), respectively, while maximum and minimum OPG was recorded in the month of September (674.05) and February (118.36), respectively. Mixed infections with more than one species of GI parasites were also recorded in 13.38% samples. The percent prevalence of different species of GI parasites in goats of Meghalaya was depicted in Figure-2. Coproculture of goat fecal samples revealed the presence of *Haemonchus contortus* (72.16%), *Oesophagostomum* spp. (14.41%), *Strongyloides* spp. (8.91%), and *Trichostrongylus* spp. (4.50%) larvae throughout the year (Table-2). Seven different species of *Eimeria* spp. were identified after examining sporulated oocysts, *viz., E. christenseni, E. hirci, E. caprina, E. jolchijevi, E. ninakohlyakimovae, E. arloingi*, and *E. kocharii* for the first time from Meghalaya (Figure-3).

**Table-1:** Season-wise prevalence of GI parasites in goats of Meghalaya.

Season	Sample examined	Sample positive	Helminths					Protozoa	Mixed infection
	
			*Strongyle* spp.	*Strongyloides* spp.	*Moniezia* spp.	*Trichuris* spp.	Overall	*Eimeria* spp.	
Hot	139	37 (26.62)	11 (29.73)	5 (13.51)	4 (10.81)	4 (10.81)	24 (64.86)	9 (24.32)	4 (10.81)
Rainy	355	124 (34.93)	45 (36.29)	15 (12.10)	14 (11.29)	8 (6.45)	82 (66.13)	23 (18.55)	19 (15.32)
Cool	134	36 (26.87)	11 (30.55)	5 (13.88)	2 (5.55)	5 (13.88)	23 (63.89)	9 (25.00)	4 (11.11)
Cold	206	42 (20.39)	11 (26.19)	5 (11.90)	4 (9.52)	3 (7.14)	23 (54.76)	14 (33.33)	5 (11.90)
Total	834	239 (28.65)	78 (32.63)	30 (12.55)	24 (10.04)	20 (8.36)	152 (63.60)	55 (23.02)	32 (13.38)

Figures in parentheses indicates percent positivity. GI = Gastrointestinal

**Table-2:** Percent composition of infective larvae in coproculture of goats.

Month	*H. contortus*	*Oesophagostomum* spp.	*Strongyloides* spp. (4.12%)	*Trichostrongylus* spp. (2.70%)
April’14	67	26	4	3
May’14	68	20	6	6
June’14	75	11	9	5
July’14	70	15	12	3
Aug’14	68	10	14	8
Sept’14	72	13	10	5
Oct’14	70	16	13	1
Nov’14	75	9	12	4
Dec’14	78	10	9	3
Jan’15	77	12	4	7
Feb’15	74	17	5	4
Mar’15	72	14	9	5
Total (%)	866 (72.16)	173 (14.41)	107 (8.91)	54 (4.50)

*H. contortus*=*Haemonchus contortus*

In the present study, the prevalence of GI parasitic infections in goats was observed throughout the year. The present finding was in accordance with Olanike *et al*. [19] and Nwigwe *et al*. [20] who reported prevalence of helminths and protozoan parasites (*Strongyle* spp.*, Strongyloides* spp., and Coccidia) in the intestinal tract of goats. After fecal culture, *H. contortus* has been reported as predominant species in goats of India [14,21]. Besides, *H. contortus*, *Trichostrongylus* spp., and *Oesophagostomum* spp. have been found as the most prevalent species of nematodes in goats [22]. The prevalence of *Eimeria* spp. (23.02%) in the present findings is in agreement with the findings of Rehman *et al*. [23] from Pakistan and Iqbal *et al*. [24] from Jammu, who reported 55.99% and 54.42% *Eimeria* spp. infection in goats, respectively. 16 species of *Eimeria* have been recorded from goats in different parts of the world [25,26]. Four species, *viz*., *E. ninakohlyakimovae*, *E. arloingi*, *E. caprina*, and *E. hirci* were identified from Pakistan [23]. Two species of coccidia, *viz*., *E. arloingi* and *E. ninakohlyakimovae* in goats have been reported from Assam [27]. However, so far our knowledge is concerned, there is no report on the prevalence of different species of *Eimeria* in goats of Meghalaya, and this report may be considered as the first report on prevalence of seven species of coccidia of goats, i.e. *E. christenseni*, *E. hirci*, *E. caprina*, *E. jolchijevi*, *E. ninakohlyakimovae*, *E. arloingi*, and *E. kocharii* from the state Meghalaya. Moreover, Iqbal *et al*. [24] observed highest infection rate in kids (74.48%) than adult goats (33.33%). The prevalence of *Eimeria* throughout the year might be due to nonadministration of coccidiostat or coccidicidal drugs by the farmers.

This shows that the climate in this region is exclusively conducive for the development and propagation of parasites. Moreover, the meteorological parameters such as temperature, humidity, and relative humidity are found to be favorable for exogenous development of GI parasites throughout the year in this region. Other factors which might be responsible are constant exposure to infections, continuous deposit of infections on the pastures by adult animals as well as poor animal husbandry practices.

## Conclusion

The present study revealed that there is prevalence of GI helminths and protozoa infections in goats of Meghalaya, throughout the year and highest during rainy season.

## Authors’ Contributions

MD and RL: Examined samples and prepared manuscript, AG: Collected, processed and examined samples, AS: Interpretation of data. All authors read and approved the final manuscript.
